# The Influence of Recombinational Processes to Induce Dormancy in *Trypanosoma cruzi*

**DOI:** 10.3389/fcimb.2020.00005

**Published:** 2020-01-28

**Authors:** Bruno Carvalho Resende, Anny Carolline Silva Oliveira, Anna Carolina Paganini Guañabens, Bruno Marçal Repolês, Verônica Santana, Priscila Mazzochi Hiraiwa, Sérgio Danilo Junho Pena, Glória Regina Franco, Andrea Mara Macedo, Erich Birelli Tahara, Stênio Perdigão Fragoso, Luciana Oliveira Andrade, Carlos Renato Machado

**Affiliations:** ^1^Laboratory of Biochemistry Genetics, Department of Biochemistry and Immunology, ICB, Universidade Federal de Minas Gerais, Belo Horizonte, Brazil; ^2^Laboratory of Cellular and Molecular Biology, Department of Morphology, ICB, Universidade Federal de Minas Gerais, Belo Horizonte, Brazil; ^3^Laboratory of Functional Genomics, Instituto Carlos Chagas, Oswaldo Cruz Foundation (FIOCRUZ), Curitiba, Brazil

**Keywords:** recombination, *Trypanosoma cruzi*, dormancy, DNA metabolism, infection

## Abstract

The protozoan *Trypanosoma cruzi* is the causative agent of Chagas disease, a neglected tropical disease that affects around 8 million people worldwide. Chagas disease can be divided into two stages: an acute stage with high parasitemia followed by a low parasitemia chronic stage. Recently, the importance of dormancy concerning drug resistance in *T. cruzi* amastigotes has been shown. Here, we quantify the percentage of dormant parasites from different *T. cruzi* DTUs during their replicative epimastigote and amastigote stages. For this study, cells of *T. cruzi* CL Brener (DTU TcVI); Bug (DTU TcV); Y (DTU TcII); and Dm28c (DTU TcI) were used. In order to determine the proliferation rate and percentage of dormancy in epimastigotes, fluorescent-labeled cells were collected every 24 h for flow cytometer analysis, and cells showing maximum fluorescence after 144 h of growth were considered dormant. For the quantification of dormant amastigotes, fluorescent-labeled trypomastigotes were used for infection of LLC-MK2 cells. The number of amastigotes per infected LLC-MK2 cell was determined, and those parasites that presented fluorescent staining after 96 h of infection were considered dormant. A higher number of dormant cells was observed in hybrid strains when compared to non-hybrid strains for both epimastigote and amastigote forms. In order to investigate, the involvement of homologous recombination in the determination of dormancy in *T. cruzi*, we treated CL Brener cells with gamma radiation, which generates DNA lesions repaired by this process. Interestingly, the dormancy percentage was increased in gamma-irradiated cells. Since, we have previously shown that naturally-occurring hybrid *T. cruzi* strains present higher transcription of RAD51—a key gene in recombination process —we also measured the percentage of dormant cells from *T. cruzi* clone CL Brener harboring single knockout for RAD51. Our results showed a significative reduction of dormant cells in this *T. cruzi* CL Brener RAD51 mutant, evidencing a role of homologous recombination in the process of dormancy in this parasite. Altogether, our data suggest the existence of an adaptive difference between *T. cruzi* strains to generate dormant cells, and that homologous recombination may be important for dormancy in this parasite.

## Introduction

The hemoflagellate *Trypanosoma cruzi* is an intracellular, protozoan parasite, and the etiological agent of Chagas disease, an infectious malady that currently affects ~8 million people worldwide. Mostly present at tropical and subtropical regions of the globe, Chagas disease is considered the most important parasitic infection in Latin America (WHO, 2019). It presents an acute phase with a relatively short duration, during which some signals and symptoms can be identified in infected individuals, such as an injury at the site of Triatominae vector bite—the chagoma or Romaña sign—and fever. Following the acute phase, the disease progresses to a chronic phase, during which is observed low parasitemia and a variable clinical course characterized by digestive, neurological, and cardiac complications (Rassi and Marin-Neto, 2010). Clinical diversity among patients has been shown to be a consequence of both host and parasite genetic variability (Vago et al., 2000). *T. cruzi* has been defined as a clonal species, but hybrid populations have also been found in nature, suggesting that events of genetic exchange may be pivotal to the genetic variability found in this parasite (Tomasini and Diosque, 2015).

Recently, *T. cruzi* strains were divided into six discrete typing units (DTUs)—TcI to TcVI—and each DTU discriminates genetically similar *T. cruzi* groups (Zingales et al., 2009). TcI occurs across North, Central, and South America—especially in Colombia and Venezuela—and is represented by strains with high replication rates and high parasitemia, leading to high mortality around 20–30 days post-infection (Zingales et al., 2012; Zingales, 2018). TcII presents higher replication and infection rates when compared to TcI, with a parasitemia peak around 12–20 days post-infection, when it promotes high mortality levels (Zingales et al., 2012; Oliveira et al., 2017). TcIII leads to low parasitemia, with its peak around 15–20 days post-infection, and is not related with chronic cases, being rarely documented in human infection (Zingales et al., 2012; Ragone et al., 2015). TcIV shows low virulence, with significant lower levels of parasitemia in comparison to TcII, but presents high prevalence in humans. Interestingly, TcV and TcVI are comprised of naturally-occurring hybrid strains resulting from genetic exchange events between TcII and TcIII. TcV and TcVI are less infective and display lower replication rates, but are largely related with severe chronic and congenital human disease. They occur mostly in South America within domestic transmission cycles (Zingales et al., 2012; Brenière et al., 2016; Oliveira et al., 2017).

In its heteroxenic cycle, *T. cruzi* metacyclic trypomastigotes—which are released together with triatomine feces—are capable of infecting different cell types from the mammalian host. Host cell infection occurs through the interaction between parasite surface and molecules from the host cell (Andrade and Andrews, 2005; Fernandes and Andrews, 2012). The internalized trypomastigotes differentiate into amastigotes, and begin replication in cytoplasm of the host cell, until they differentiate back into trypomastigotes. Extracellular-released trypomastigotes may infect neighboring cells, or even reach the bloodstream, and spread through the whole organism infecting other tissues (De Souza, 1999).

Recently, Sánchez-Valdéz et al. (2018) described the occurrence of cellular dormancy in *T. cruzi* during *in vivo* and *in vitro* experimental infection. Dormancy is a state involved in resistance to non-optimal environmental conditions of life in which cells become arrested. Dormancy has been described in various organisms: in fungi, it has been shown to avoid spore germination in harmful conditions (Wyatt et al., 2013); in bacteria, dormancy is well-known to occur in order to preserve the ability of colonizing the host even after the exposure to antibiotics (Harms et al., 2016). Cancerous cells can also undergo a dormant state upon exposure to chemotherapeutic agents aimed to eliminate replicative, active cells (Naumov et al., 2001). In protozoa, dormancy has been characterized in *Plasmodium* sp., the causative agent of malaria. In *Plasmodium*, the dormant form is recognized as a specific stage, called hypnozoite, and is associated with the recurrence of the disease and drug resistance (Markus, 2012). For *T. cruzi*, it was demonstrated that replicative-arrested amastigotes failed to incorporate nucleotide analog 5-ethynyl-2′-deoxyuridine (EdU), but were able to perform differentiation into trypomastigotes. Interestingly, those dormant parasites were resistant to doses of benznidazole 50-fold higher than the regular IC_50_ dose. Dormant cells recovered growth after 30 days post-infection (Sánchez-Valdéz et al., 2018).

Our group has shown through different works that homologous recombination is essential in *T. cruzi* once it allows this parasite to repair DNA double strand breaks (DSBs), as well as it is also implicated in genetic exchange between *T. cruzi* cells (Alves et al., 2018). We have also shown that this parasite survives up to 500 Gy of gamma radiation—which induces DSBs—repairing DNA lesions within 48 h, and recovering cell growth 96 h after irradiation (Regis-da-Silva et al., 2006). Noteworthy, TcRad51, a protein involved in homologous recombination, has demonstrated to play a major role in recombinational process in *T. cruzi*. RAD51 is involved in the process of double strand DNA invasion through homology, forming a D-loop which will be processed by other proteins involved in this pathway (Holloman, 2011). *T. cruzi* overexpressing TcRAD51 is capable of resolving DSBs in both epimastigotes and amastigotes. On the other hand, TcRAD51-deficient epimastigotes and amastigotes are unable to properly repair DSBs (Gomes Passos Silva et al., 2018). Homologous recombination and TcRad51 also play a relevant role in genetic exchange processes since the overexpression of TcRAD51 is capable of stabilizing hybrid *T. cruzi* cells. In fact, naturally-hybrid *T. cruzi*'s populations show higher expression of TcRAD51 when compared to the non-hybrid ones (Alves et al., 2018).

In the present work, we used CellTrace™ CFSE Cell Proliferation Kit to assess *T. cruzi* asynchronous replication, focusing on dormant, replicatively-arrested cells in epimastigotes and amastigotes from different *T. cruzi* groups, as well as in parasites exposed to gamma radiation or deficient in TcRAD51. We verified that naturally-occurring hybrid cells—which express more TcRAD51—present a higher percentage of dormant epimastigotes and amastigotes when compared to non-hybrid ones. In epimastigotes, gamma radiation was able to increase dormancy, while CL Brener TcRAD51 single knockout cells (CL Brener^RAD51+/−^) presented decreased dormancy when compared to wild-type cells (CL Brener^WT^). Altogether, our data suggest that homologous recombination may play a key role in dormancy signaling.

## Materials and Methods

### Cell Types and Cellular Culture

*T. cruzi* epimastigotes were maintained at 28°C in liver infusion tryptose (LIT) medium supplemented with 10% inactivated fetal bovine serum (Gibco), 200 units/mL penicillin, and 200 μg/L streptomycin sulfate. *T. cruzi* clones CL Brener and Y were provided by Dr. Egler Chiari (Universidade Federal de Minas Gerais); clone Bug was provided by Dr. Bianca Zingales (Universidade de São Paulo); and clone Dm28c was provided by Dr. Stenio Fragoso (Fiocruz—Paraná). CL Brener^RAD51+/−^ were generated by Gomes Passos Silva et al. (2018). Rhesus monkey kidney cells (LLC-MK2; Hull et al., 1962) monolayers were maintained in 10% Dulbecco's Modified Eagle's Medium (10% DMEM; Sigma Aldrich), containing 10% FBS, 200 units/mL penicillin, and 200 μg/L streptomycin sulfate. Metacyclic trypomastigotes obtained from axenic cultures of *T. cruzi* at stationary phase were used to initiate parasite intracellular life cycle in LLC-MK2 cells. Infection was performed in 2% DMEM (2% FBS, 200 units/mL penicillin, and 200 μg/L streptomycin sulfate). LLC-MK2 culture was washed on a daily basis with PBS buffer containing Ca^2+^ and Mg^2+^ (PBS^+/+^) to remove epimastigotes from the medium. Released tissue-culture trypomastigotes (TCTs) were purified as described previously (Andrews et al., 1987), and used to maintain parasite intracellular life cycle and to perform all experiments involving these cells.

### CFSE Labeling and Sorting

Populations of *T. cruzi* epimastigotes were labeled with CellTrace CFSE (ThermoFisher) following the manufacturer instructions. Briefly, 2 × 10^7^ epimastigotes/mL were incubated for 20 min at 28°C with 10 mM CFSE in PBS buffer, protected from light. The excess of CFSE was quenched with 5 volumes of LIT medium for 5 min. After that, cells were centrifuged at 3,000 × *g* for 10 min and resuspended in fresh LIT medium at the concentration of 1 × 10^7^ cells/mL. Aliquots from CFSE-stained epimastigote cultures were collected during 144 h, counted in a cytometry chamber and analyzed by flow cytometry to assess fluorescence intensity and replication profile. For some experiments, parasites were irradiated with a dose of 1,541 Gy/h for 19 min and 28 s using a cobalt (^60^C) irradiator located at Laboratório de Irradiação Gama (CDTN/CNEN, UFMG), prior to CFSE labeling and culture growth. FACSCan or FACSCalibur flow cytometers were used for data collection (Benckton-Dickson) and 10,000 events for each condition were analyzed using the software FlowJo VX. FACSAria (Benckton-Dickson) sorter was used to create an enriched population of epimastigotes that had not replicated since the beginning of the analyses (24 h) until the stationary phase (144 h).

### Mammalian Cell Infection and Immunostaining

For cell infections 4 × 10^4^ LLC-MK2 cells were suspended in 10% DMEM and added onto 13 mm round glass coverslips inserted into each well of a 24-well plate. Plated cells were then incubated at 37°C with 5% CO_2_ for 24 h before infection with purified trypomastigotes from Dm28c, Y, CL Brener^WT^, CL Brener^RAD51+/−^, or Bug strains previously labeled with CellTrace CFSE. For trypomastigote CFSE labeling, parasites were incubated for 20 min at 37°C with 10 mM CFSE in PBS buffer. The excess of CFSE was quenched with 2% DMEM for 5 min. After that, cells were centrifuged at 3,000 × *g* for 10 min and resuspended with 5 volumes of 2% DMEM. Infection was performed (protected from light) at a multiplicity of infection (MOI) of 50 for 1 h in 2% DMEM. Afterwards, cells were washed five times with PBS^+/+^ and re-incubated in 2% DMEM for additional 24, 48, 7, and 96 h, until overnight fixation with 4% PFA at 4°C, temperature which samples were stored until processed for immunofluorescence.

After fixation, coverslips with attached cells were washed three times with PBS^+/+^ in order to remove cell fixative, incubated for 20 min with PBS containing 2% BSA (PBS/BSA) and processed for an inside/outside immunofluorescence invasion assay as previously described (Andrews et al., 1987). Briefly, extracellular parasites were immunostained with rabbit anti-*T. cruzi* polyclonal antibodies (Andrade and Andrews, 2004) in a 1:500 dilution in PBS/BSA for 1 h at room temperature, washed and labeled with Alexa Fluor-546 conjugated anti-rabbit IgG antibody (Thermo Fischer Scientific) in a proportion of 1:500 in PBS/BSA for 45 min. After that, DNA from host cells and parasites was stained for 1 min with 0.1 μM DAPI (4′,6-Diamidino-2-Phenylindole, Dihydrochloride—Sigma) in PBS, mounted, and examined on a Zeiss Axio Vert.A1 microscope equipped with an AXIOCAM ICM1 camera controlled by the ZEN Image Software (Zeiss).

### RNA Extraction and cDNA Synthesis

*T. cruzi* total RNA was extracted from 1 × 10^8^ cells using TRIzol (Invitrogen) according to the manufacturer recommendations. After RNA extraction, contaminant DNA was removed from samples through the use of TURBO DNA-Free Kit (Ambion, ThermoFisher). In order to verify possible persistence of contaminant DNA, each sample was submitted to a PCR carried out using the primers listed in Table 1. Treatment with TURBO DNA-free Kit was repeated until PCR was negative. A total of 1 μg of clean RNA were used for cDNA synthesis reaction using the High Capacity cDNA Reverse Transcription Kit (Life Technologies). As negative control, a reaction with no reverse transcriptase was carried out.

**Table 1:** Primers used on Real-time PCR quantification.

**Primer**	**Sequence**
RTTcRad51Fw	5′-GGC TGT CAA GGG TAT CAG TG−3′
RTTcRad51Rev	5′-AAC CAC TGC GGA TGT AA GC−3′
RTGAPDH2Fw	5′-CGGTGGACGGTGTGTCGGTG-3′
RTGAPDH2Rev	5′-CCGTCAGCTTGCCCTGGGTG-3′

### Quantitative Real-Time PCR

Reactions were performed using SYBR Green PCR Master Mix (Applied Biosystems). For each reaction, 10 ng cDNA, 300 nM primers, 5 μL SYBR Green Master Mix 2X, and DNAse/RNAse-free water—for a total volume of 10 μL of reaction—were used. Reactions were performed in a 384-well plate in technical and biological triplicates. Glyceraldehyde 3-phosphate dehydrogenase gene (GAPDH) was used as a loading control. All primers used in the quantification experiments are listed in Table 1. The relative amount of TcRAD51 transcripts were calculated by the 2^−ΔΔCt^ method.

### Statistical Analysis

Statistical analyses were performed using GraphPad Prism 5 version 5.01 (San Diego, USA). Data were analyzed through one-way ANOVA with Bonferroni Comparison Test as post-test. *p* < 0.05 was reported as significant.

## Results

### Epimastigote Dormancy Is Strain-Dependent in *T. cruzi*

**Figure 1:**
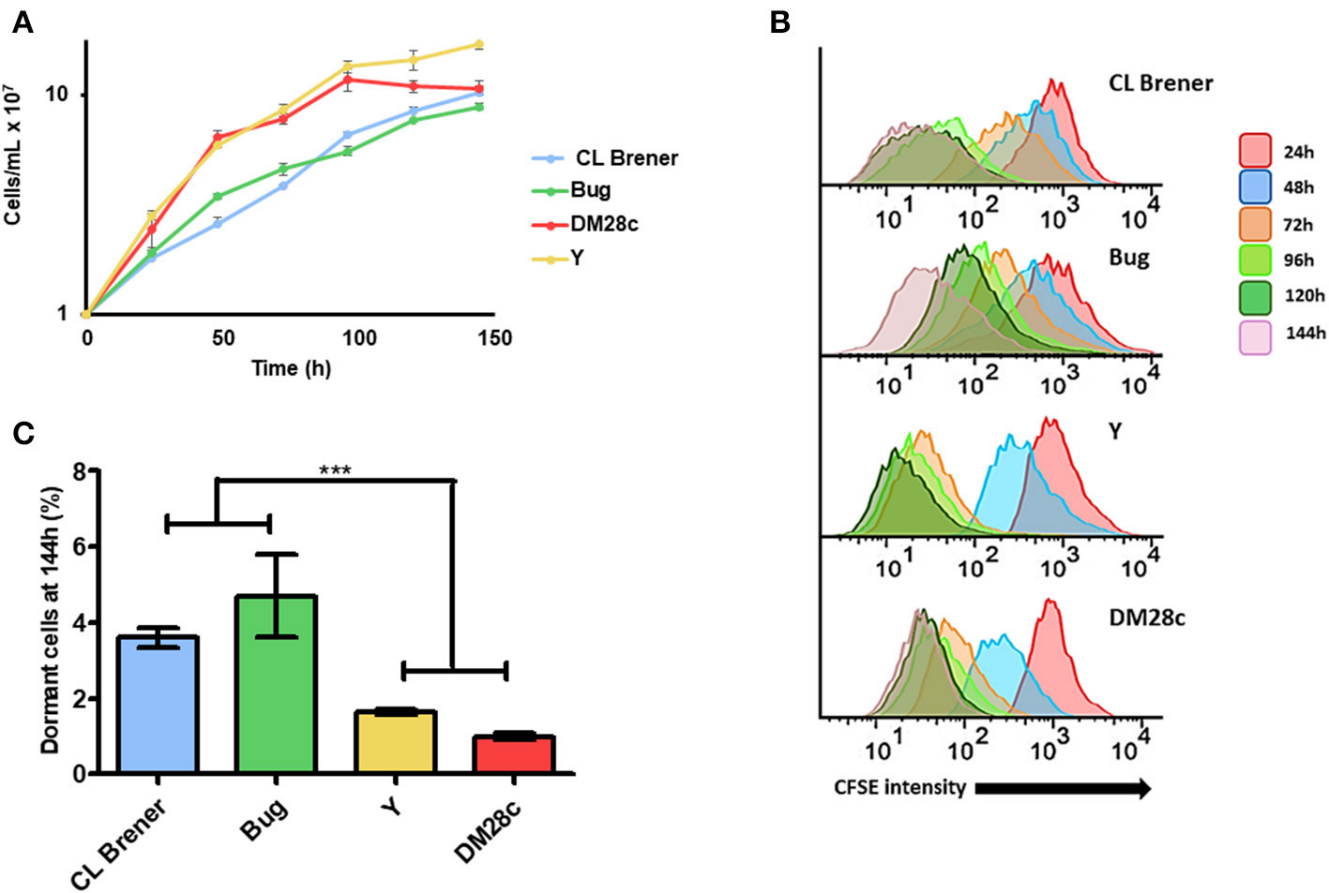
Dormancy in epimastigote forms of *T. cruzi* wild type strains. **(A)** Cellular growth curves from four different strains (CL Brener, Bug, Dm28c, and Y). At 0 h, 1 × 10^7^ cells were treated with CFSE and samples were counted every 24 h for 144 h. **(B)** Flow cytometry histograms of epimastigote cultures from each strain from 24 to 144 h. CFSE intensity was assessed every 24 h until 144 h, and arrested cells were considered those ones which exhibited similar CFSE intensity at 144 h when compared to the level of half median of 24 h. **(C)** Average of percentage of dormant cells in CL Brener, Bug, Y, and Dm28c strains at 144 h was detected in flow cytometry histograms. Representative results of three experiments. Asterisks indicate statistically significant differences among groups (*P*-value <0.05).

In order to evaluate if epimastigote dormancy is related to the genetic background in *T. cruzi*, we tested cells from four different DTUs: Dm28c (TcI), Y (TcII), CL Brener (TcVI), and Bug (TcV). For such, we labeled epimastigotes from all aforementioned strains with CellTrace™ CFSE Cell Proliferation Kit (Item 2.2), and followed its fluorescence decay during 144 h of culture through flow cytometry. Cellular duplication should reduce CFSE fluorescence intensity by 0.55-fold after each cycle, indicating that cells whose CFSE fluorescence intensity remains at high levels over time can be considered as dormant. Fluorescence intensity at the very beginning of cellular culture (0 h) was not considered for analysis in order to avoid biased interpretations due to an inevitable loss of CFSE right after cellular labeling. After 144 h of monitoring, we observed that naturally-occurring hybrid strains CL Brener and Bug showed a lower growth rate when compared to non-hybrid strains Dm28c and Y (Figure 1A). Interestingly, we also verified that all strains studied present arrested parasites in all time points analyzed. However, we also observed that CL Brener and Bug display an increased percentage of arrested parasites compared to Dm28c and Y during the 144 h of culture (Figures 1B,C). In fact, CL Brener and Bug consistently presented a higher percentage of dormant cells since 24 h of culture through the end of the experiment (Figure 1B). Altogether, these results indicate that *T. cruzi* epimastigotes from naturally-occurring hybrid strains exhibit an increased ability to enter in dormancy.

### Dormant Epimastigotes Can Resume Cellular Duplication in Fresh Conditions

**Figure 2:**
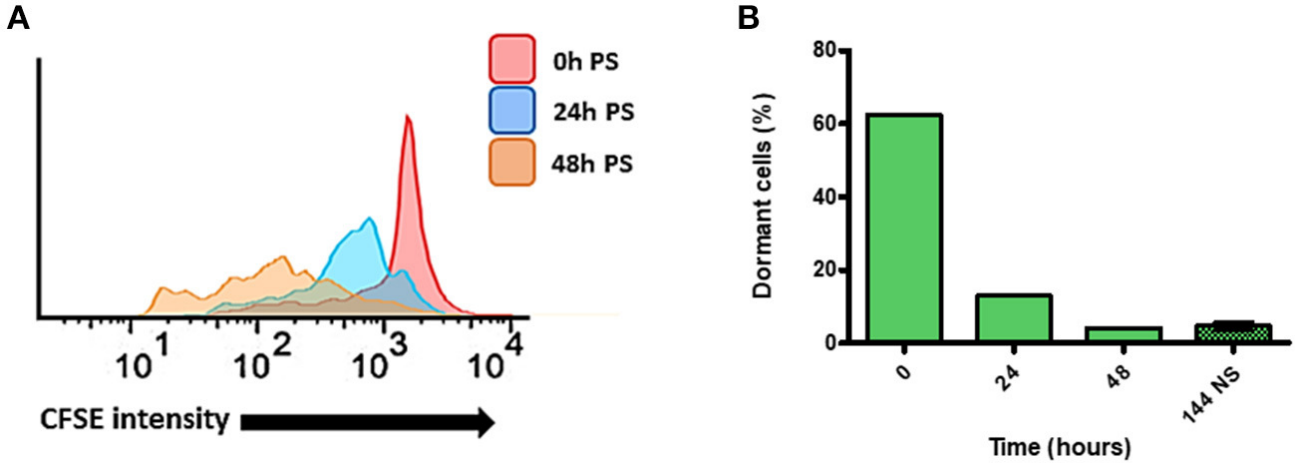
Quantification of dormant replication cells after sorting of dormant epimastigote cells. **(A)** Flow cytometry histograms of dormant epimastigotes of Bug strain after sorting. Cells sorted (0 h post-sorted; PS) were resuspended in fresh medium, and CFSE intensity was followed every 24 h until 48 h PS. Cells that exhibited similar CFSE intensity at each point when compared to the level of half median of 0 h PS were considered arrested cells. **(B)** The percentage of arrested cells after sorting as detected in flow cytometry histograms in each time point. The percentage of dormant cell before sorting at 144 h is presented to comparation.

In order to verify, if arrested *T. cruzi* cells can resume their cellular duplication and, if so, what is the pattern of growth wherewith it occurs, we used the cell sorter FACSAria (Beckton-Dickson) to obtain a population enriched in dormant epimastigotes from Bug strain—as it presented the highest percentage of dormant cells from all strains studied (Figure 1). For such, Bug epimastigotes exhibiting a sustained CFSE fluorescence intensity at 144 h (half the median at 24 h point) culture were sorted by FACSAria in fresh LIT, allowing an enrichment of 1,550% (62% of dormant cells after sorting vs. 4% of dormant cells before sorting) (Figure 2A). CFSE fluorescence intensity decay of this population was then followed over time. We verified that in all time points analyzed (24 and 48 h), CFSE intensity progressively decreased, indicating cellular duplication (Figures 2A,B). Interestingly, we also noticed that the percentage of dormant epimastigotes 48 h post-sorting and growth was similar to that one observed for Bug epimastigote cultures at 144 h of growth pre-sorting—around 4% (Figure 2B).

### Amastigote Dormancy Is Also Strain-Dependent in *T. cruzi*

**Figure 3:**
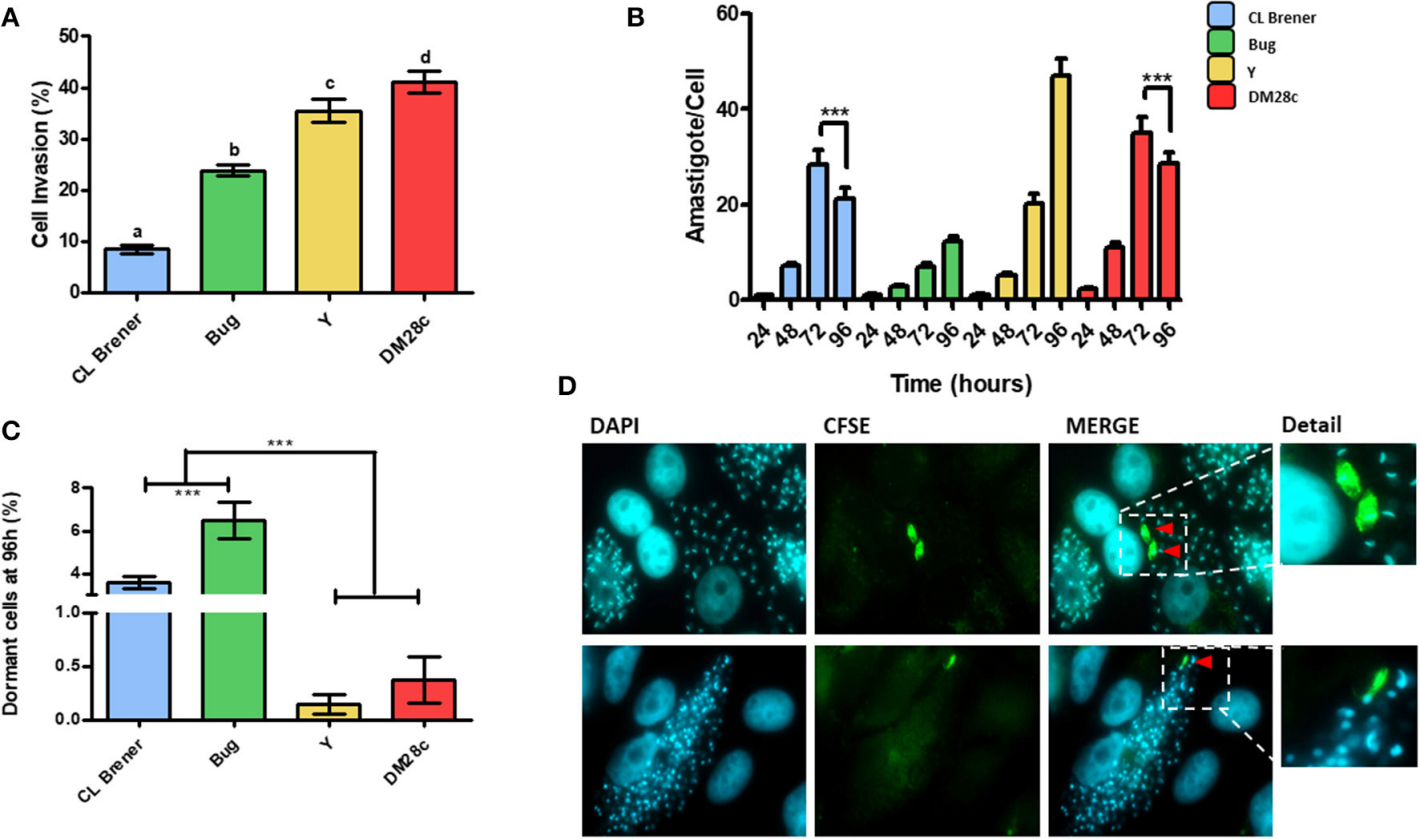
Dormancy in amastigote forms of *T. cruzi*. **(A)** Invasion rate of CL Brener, Bug, Y, and Dm28c strains in LLC-MK2 cells. The graph shows the percentage of infected LLC-MK2 cells in a total of 250 cells were analyzed (parasites labeled with anti-*T. cruzi* antibody were disregarded). Letters indicate statistically significant differences among each strain (*P*-value <0.05). **(B)** *T. cruzi* infection progression. The graph shows the number of intracellular parasites per infected cells. A total of 100 infected LLC-MK2 cells were analyzed at each time point. Asterisks represent the statistically significant differences at 96 h post infection in CL Brener and Dm28c when compared to 72 h post infection (*P*-value <0.05). **(C)** The graph shows the percentage of intracellular amastigotes in a dormant state 96 h post-infection. A total of 100 LLC-MK2 infected cells were analyzed. Asterisks indicate statistically significant differences among groups (*P*-value <0.05). **(D)** Representative images of DM28c infected cultures, 96 h post-infection. Red arrows indicate dormant (CFSE+) amastigotes (upper panel) and CFSE positive trypomastigotes (lower panel) (100x). The CFSE+ amastigote and trypomastigote form were detailed. Representative results of two distinct experiments in three technical repetitions repeats.

Since duplication rates and the percentage of dormant cells in *T. cruzi* epimastigotes were dependent on the genetic background (Figure 1), we hypothesized that this dependency could be also observed for amastigotes. We then performed infection of LLC-MK2 cells using trypomastigotes from Dm28c, Y, CL Brener, and Bug strains, and followed their intracellular transformation into amastigotes and replication of the latter for a period of 96 h of infection. The percentage of infected LLC-MK2 cells and the percentage of CFSE-positive amastigotes were evaluated. The latter was evaluated by determining the number of CFSE-labeled from the total amastigotes found in 100 LLC-MK2 infected cells. Amastigotes presenting high CFSE fluorescence intensity 96 h post-infection were considered arrested. We observed that the invasion rate of LLC-MK2 cells by CL Brener and Bug—naturally-occurring hybrids—was lower in relation to the invasion rate observed for Dm28c and Y—non-hybrid strains (Figure 3A). Interestingly, the highest number of total Dm28c and CL Brener amastigotes (i.e., CSFE-positive and negative amastigotes) per cell was observed 72 h post-infection, with a reduction in this number 96 h post-infection (Figure 3B). This decline is likely related to the fact that for these strains about 4% of the cells (3.3% in CL Brener strain, and 4.3% in DM28c strain) were infected with only one parasite 96 h post-infection.

Ninety-six hours post-infection, most amastigotes present in LLC-MK2 cells were CFSE-negative due to fluorescence decay promoted by cellular replication. Therefore, at this time point, CFSE-positive amastigotes represent those parasites which underwent few duplication events, and thus can be classified as dormant cells. Interestingly, we verified that the percentage of dormant amastigotes from CL Brener and Bug was higher in relation to Dm28c and Y (Figure 3C), in similar fashion to the results obtained with epimastigotes (Figure 1). Finally, it is noteworthy that CFSE-positive trypomastigotes were found inside LLC-MK2 cells at 96 h pot infection (Figure 3D), strongly suggesting that they had derived from dormant amastigotes.

### Dormant Amastigotes Can Be Converted Into Infecting Trypomastigotes

**Figure 4:**
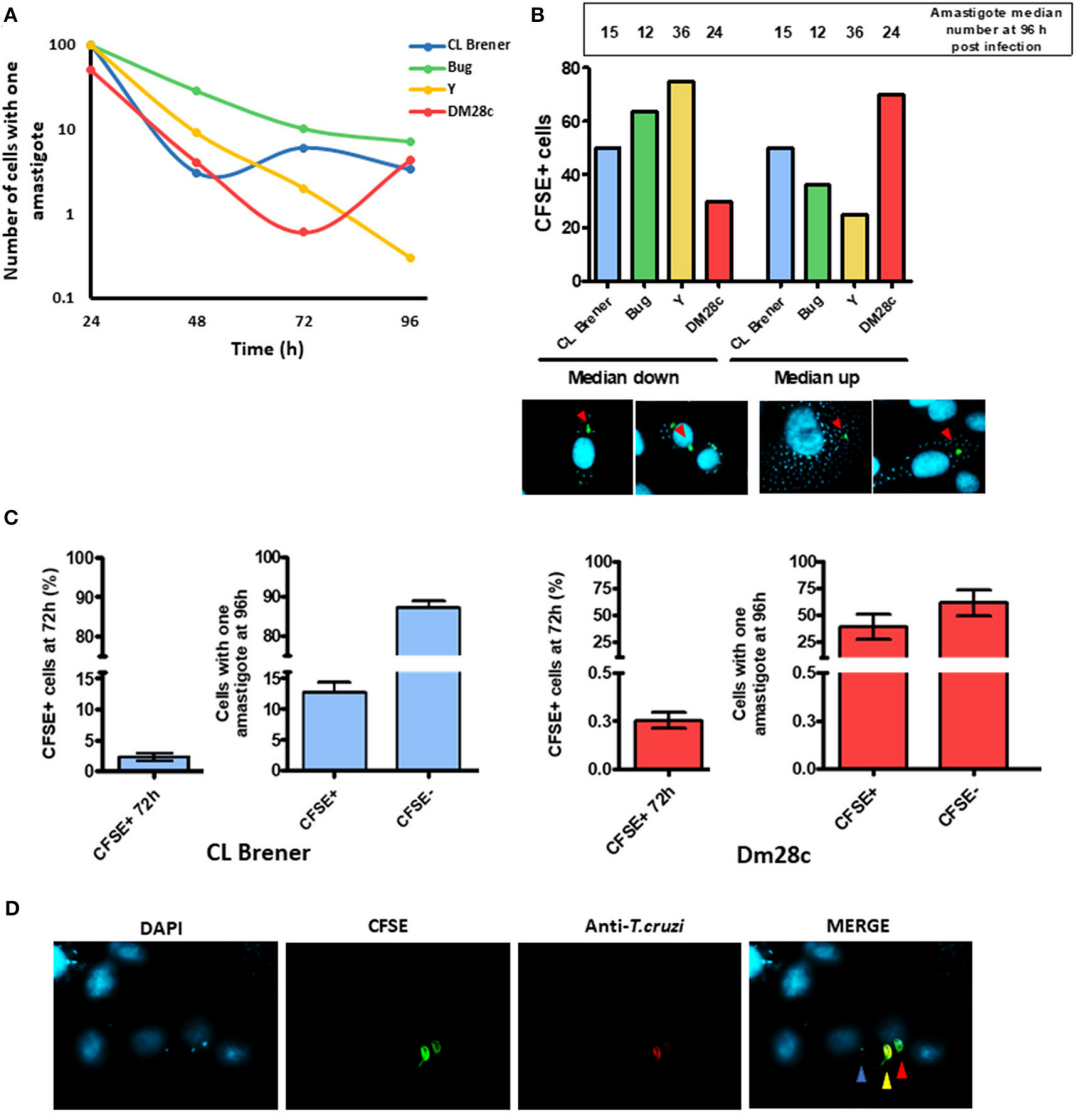
Dormant reinfection characterization. **(A)** The graph shows the percentage of cells infected with a single amastigote along the infection. A total of 100 infected LLC-MK2 cells were analyzed at each time point and the infected cells with just one amastigote were counted. **(B)** The graph shows the percentage of cells containing CFSE-positive parasites according two groups of cells: one in which the number of intracellular parasites was higher than the median, and another one in which the number was lower than the median at 96 h post-infection. Values indicated in the bar are the median for each strain at 96 h post-infection. Below the graph, a representative figure depicting what was considered as cells with CFSE-positive parasites in a median down group or in median up group is shown. **(C)** The graph shows the percentage of cells infected with a single parasite from CL Brener or Dm28c strains at 96 h that is either CFSE-positive or -negative. A hundred single-infected cells were analyzed per assay. For the sake of comparison, besides each graphic the percentage of total CFSE positive CL Brener or Dm28c parasites at 72 h post-infection is shown. **(D)** Representative image of a cell infected with a single parasite (Dm28c strain), CFSE+ (red arrow) or CFSE- parasite (blue arrow) at 96 h post-infection. An external trypomastigote form is also shown (yellow arrow).

The high prevalence of LLC-MK2 cells harboring one amastigote 96 h post-infection (Figure 3) led us to ask if those cells were newly-infected or if the single hosted-amastigote had not been capable of forming an amastigote nest. We then sought to investigate the replication dynamics of trypomastigote-derived amastigotes in LLC-MK2. We found that, 24 h post infection, 50% of Dm28c-infected LLC-MK2 cells presented more than one amastigote, while infection carried out with Y, CL Brener, and Bug led to 100% of LLC-MK2 infected cells harboring a single amastigote (Figure 4A); this suggests that, in the very beginning of the infection, Dm28c replicates at a higher rate when compared to the other strains studied. Another observation is that the percentage of LLC-MK2 cells carrying a single amastigote decreases over time (Figure 4A).

Since the Dm28c strain exhibited a higher replication rate, we further evaluated whether arrested Dm28c amastigotes could be detected earlier in relation to arrested amastigotes from Y, CL Brener, and Bug strains. For such, we assumed that the earlier the CFSE-labeled amastigotes become arrested, the lower should be their number in relation to CFSE-negative amastigotes at later time points—e.g., 96 h post infection. We then determined the median of parasites per infected LLC-MK2 cell 96 h post-infection for each strain, and further divided the LLC-MK2 cells into two groups: one in which the number of infecting amastigotes was higher than the median (group #1), and another one in which the number of infecting amastigotes was lower than the median (group #2). For the Dm28c strain, CFSE-positive amastigotes were mostly found in LLC-MK2 cells from group #1, and for Y and Bug strains, CFSE-positive amastigotes were mostly found in LLC-MK2 cells from group #2. The CL Brener strain exhibited the same percentage of CFSE-positive amastigotes in both groups #1 and #2 (Figure 4B).

One general observation is that the number of LLC-MK2 cells infected with one amastigote decreases over time; however, infection with Dm28c and CL Brener strains leads to an increase in the number of LLC-MK2 cells infected with just one parasite at later times of infection (Figure 4A). For Dm28c, the percentage of LLC-MK2 cells with one amastigote rises from 0.6% (72 h post-infection) to 4.3% (96 h post-infection), and for CL Brener it increases from 3% (48 h post-infection) to 6% (72 h post-infection) (Figure 4A). These observations suggest that there is a phenomenon of reinfection taking place at these time intervals. We further evaluated the percentage of LLC-MK2 cells infected with a single amastigote, in this case CFSE-positive or CFSE-negative, at 96 h post-infection. We verified that the percentage of CFSE-positive CL Brener amastigotes in cells infected with just one parasite was 15% at 96 h post-infection (Figure 4C). This number means a large increase in CFSE positive cells, since within 72 h CFSE positive parasites represent only 2% of all parasites present. For Dm28c, these parasites represented 1% (of all parasites present) and 40% (in cells infected with only one parasite), for 72 and 96 h post-infection, respectively (Figures 4C,D). These results suggest that arrested parasites are more prone to reinfection than non-arrested ones.

### Homologous Recombination Induces Dormancy in *T. cruzi* Epimastigotes

**Figure 5:**
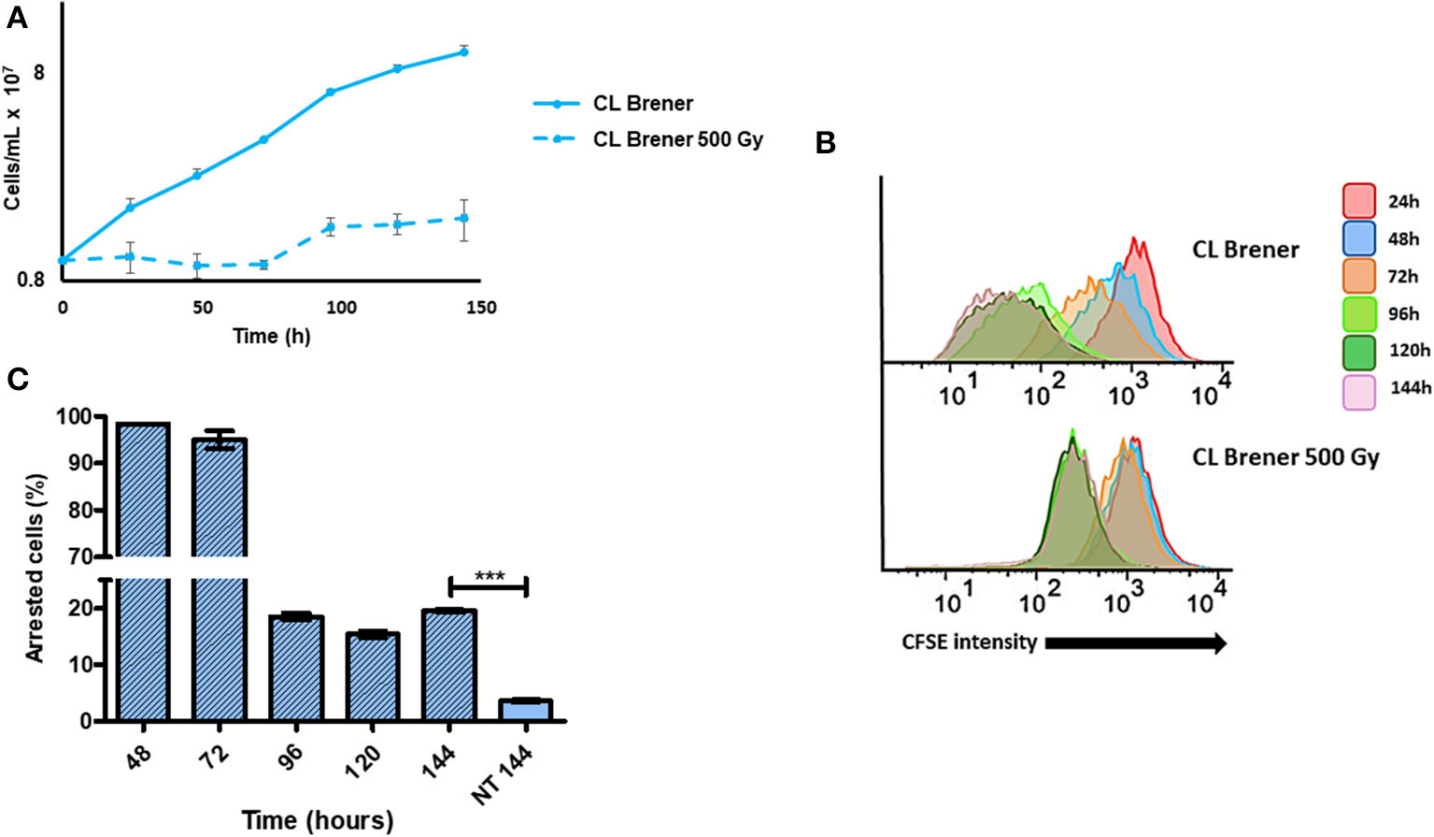
Dormancy in epimastigotes from CL Brener strain after gamma radiation exposure. **(A)** A 144 h-growth curve of wild-type *T. cruzi* CL Brener strain exposed or not to 500 Gy of gamma irradiation. **(B)** Flow cytometry histograms of CFSE fluorescence intensity decay over time from epimastigotes exposed or not to 500 Gy. Cells that have similar CFSE fluorescence intensity compared to the level of half median of 24 h time point were considered as arrested. **(C)** The graph shows the percentage of arrested parasites determined by flow cytometry showing half of CFSE median intensity observed at 24 h of growth. The percentage of dormant parasites at 144 h- from a culture without exposure to gamma radiation (NT 144 h) is shown for the sake of comparison. Representative results of three distinct experiments are shown. Asterisks indicate statistically significant differences among groups (*P*-value <0.05).

Gamma radiation generates DSBs in DNA, which are repaired by homologous recombination in *T. cruzi*. We have previously shown that epimastigotes exposed to 500 Gy of gamma radiation enter in a cell cycle arrest for 4 days, by the end of which are able to resume their cellular growth (Gomes Passos Silva et al., 2018). In order to analyze in detail how gamma radiation affects epimastigotes of *T. cruzi* from CL Brener strain, we irradiated cells with 500 Gy of gamma rays and labeled them immediately with CFSE. As expected, cellular growth recovery was observed at 96 h post-radiation, with a consequent decay in CFSE fluorescence intensity (Figures 5A,B). However, we also verified that the percentage of arrested epimastigotes at 144 h of culture was significantly higher for gamma ray-treated epimastigotes when compared to non-treated ones at same time points (Figures 5B,C). These data suggest that DNA repair through homologous recombination elicited by DSBs induces arrested cells in *T. cruzi* epimastigotes.

### RAD51 mRNA Levels Are Directly Correlated With Dormancy in *T. cruzi*

**Figure 6:**
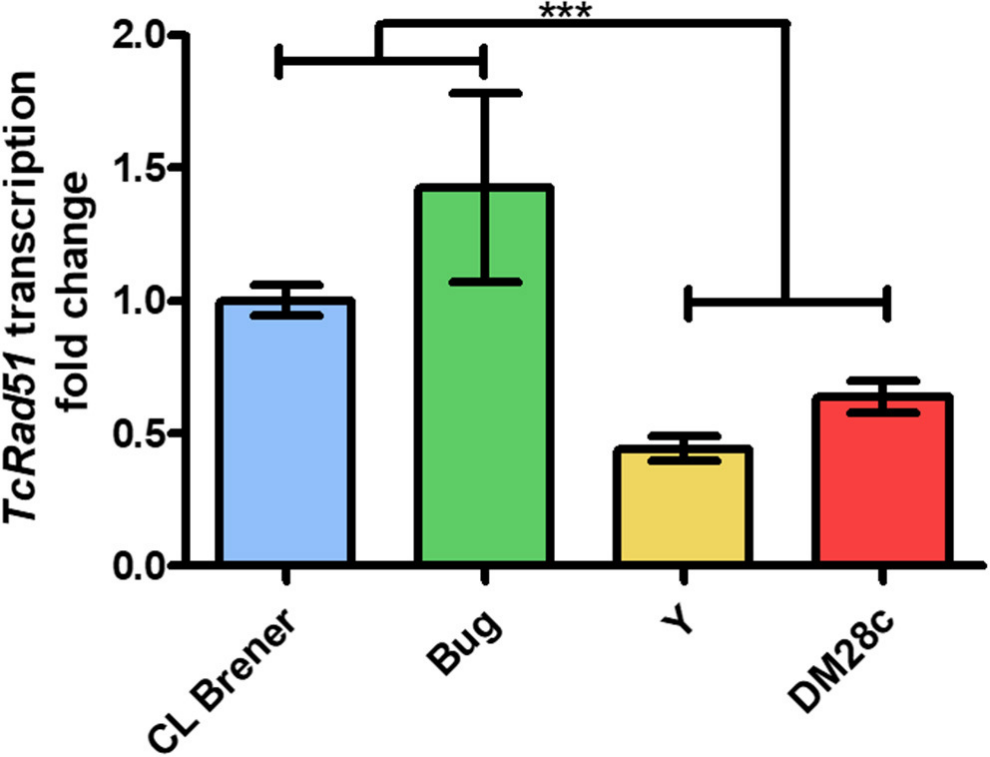
Relative expression of TcRAD51. Fold change of TcRAD51 transcription using CL Brener strain as control strain using qRT-PCR assay. Results of three distinct experiments are shown. Asterisks indicate statistically significant differences among groups (*P*-value <0.05).

We have described previously that distinct responses to DSBs, through homologous recombination, observed in different *T. cruzi* DTUs are related to their variable TcRAD51 transcription levels (Alves et al., 2018). Then, in order to verify if there is a direct relation between TcRAD51 mRNA levels and the rate of arrested cells in the strains studied here—Dm28c, Y, CL Brener, and Bug—we performed a qRT-PCR assay to determine the TcRAD51 transcription levels. We then verified that the relative TcRAD51 transcription levels of Dm28c, Y and Bug strains vary (0.64 ± 0.10, 0.45 ± 0.08, and 1.43 ± 0.62, respectively) when compared to the CL Berner strain (Figure 6). In fact, those variations show that non-hybrid strains—i.e., Dm28c and Y—display decreased TcRAD51 mRNA levels in relation to the CL Brener strain—a naturally-occurring hybrid. In line with this rationale, another naturally-occurring hybrid strain—Bug—exhibit increased TcRAD51 transcription when compared to the non-hybrid strains studied, being surprisingly higher when also compared to the CL Brener TcRAD51 mRNA levels.

### CL Brener^RAD51+/-^ Epimastigotes Exhibit Decreased Dormancy

**Figure 7:**
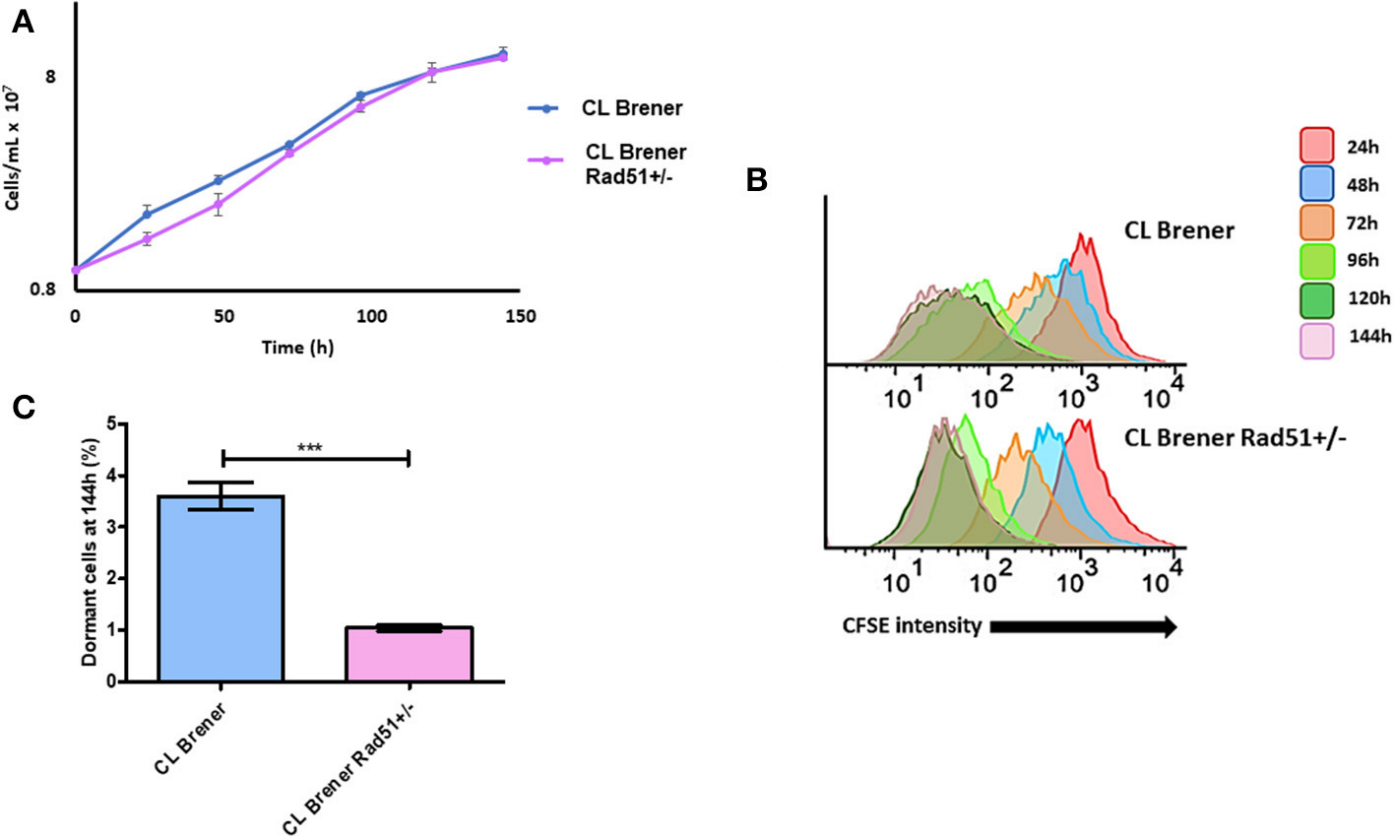
Dormancy in epimastigote forms of *T. cruzi* CL Brener Rad51^+/−^. **(A)** Cellular growth curve of CL Brener wild-type strain and Rad51^+/−^ mutant. At 0 h, 1 × 10^7^ cells were treated with CFSE, and samples were analyzed each 24 h for 144 h. **(B)** Flow cytometry histograms of epimastigote cultures from each strain. CFSE fluorescence intensity was followed every 24 h until 144 h. Cells that exhibited similar CFSE fluorescence intensity at 144 h when compared to the level of half median of 24 h were considered as dormants. **(C)** Average of dormant cells percentages in CL Brener and Rad51^+/−^ mutants at 144 h was detected in flow cytometry histograms. Representative results of three experiments are shown. Asterisks indicate statistically significant differences among groups (*P*-value <0.05).

Since increased TcRAD51 transcription levels are directly related to higher dormancy in *T. cruzi* (Figure 6), we hypothesized that the CL Brener^RAD51+/−^ mutant, which harbors only one copy of TcRAD51 (Gomes Passos Silva et al., 2018), would exhibit a decreased percentage of arrested parasites when compared to CL Brener^WT^. In fact, epimastigote cultures of CL Brener^RAD51+/−^ showed a decreased percentage of cells presenting high CFSE fluorescence intensity, indicating a high duplication rate and a reduced percentage of dormant cells when compared to CL Brener^WT^ parasites (Figures 7B,C). Interestingly, the reduction in the number of arrested parasites for CL Brener^RAD51+/−^ did not alter their rate of growth (Figure 7B), despite the fact that CFSE fluorescence intensity decay is different between CL Brener^WT^ cells and CL Brener^RAD51+/−^ mutants (Figure 7B).

### CL Brener^RAD51+/-^ Mutants Exhibit Changes in Infectivity, Replication, and Morphology

**Figure 8:**
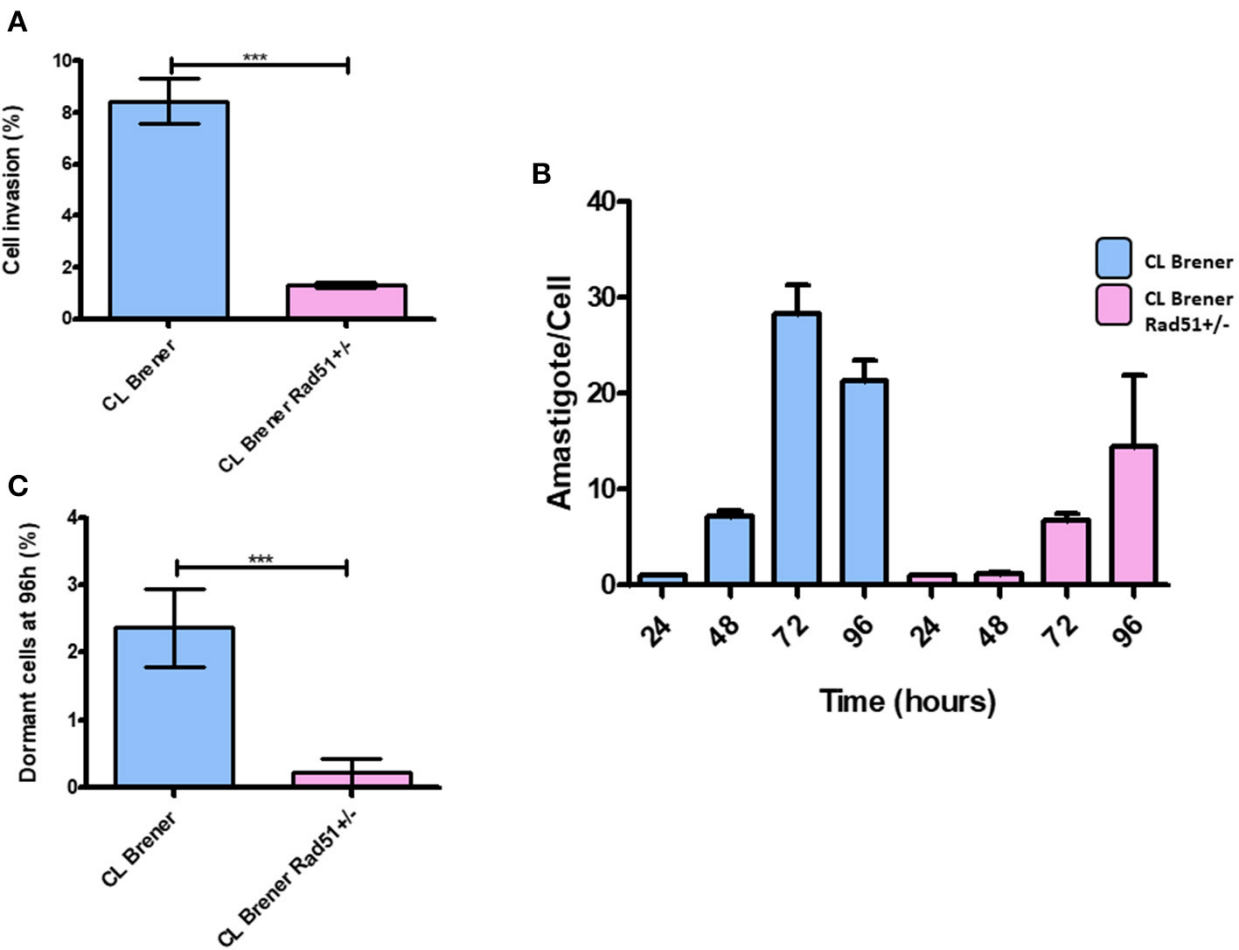
Dormancy in amastigote forms of *T. cruzi* CL Brener Rad51^+/−^. **(A)** The graph shows the percentage of infected cells in LLC-MK2 cultures infected by *T. cruzi* CL Brener Rad51^+/−^ mutants and WT cells. The number of 250 LLC-MK2 cells were analyzed (parasites labeled with anti-*T. cruzi* antibody were disregarded). Asterisk indicate statistically significant differences between strains (*P*-value <0.05). **(B)** The graph shows the rate of intracellular parasitic replication in LLC-MK2 cultures exposed to *T. cruzi* CL Brener Rad51^+/−^ mutants and WT cells during 96 h of parasite exposure. A total of 100 infected LLC-MK2 cells were analyzed at each time point. **(C)** Percentage of intracellular parasites from *T. cruzi* CL Brener Rad51^+/−^ mutants and WT cells showing high CFSE labeling 96 h post-infection. One hundred infected LLC-MK2 cells were analyzed, and the percentage of CFSE-positive ones in relation to the total number of intracellular parasites was determined.

In order to further investigate the impact of single deletion of TcRAD51 in *T. cruzi* on infectivity and replication, we carried out infection of LLC-MK2 cells using CL Brener^RAD51+/−^ and CL Brener^WT^ trypomastigotes. We observed that CL Brener^RAD51+/−^ mutants were less efficient to invade the cells than their WT counterparts (Figure 8A). As previously reported by Alves et al. (2018), CL Brener^RAD51+/−^ cells also showed a decreased replication rate in LLC-MK2 cells (Figure 8B). Nonetheless, the percentage of CFSE-positive parasites 96 h post-infection was also lower in CL Brener^RAD51+/−^ than in CL Brener^WT^ (Figure 8C).

## Discussion

In 2018, Sánchez-Valdéz et al. first showed that *T. cruzi* has the ability to enter into a dormant state as they observed that some amastigotes interrupted their cellular replication during an *in vitro* infection using Vero cells (Sánchez-Valdéz et al., 2018). In this work, we sought to further investigate whether *T. cruzi* dormancy also occurs in epimastigotes, as well as if dormancy is a strain-dependent process in this parasite. In order to monitor *T. cruzi* replication, we used CFSE, a compound that covalently binds to lysine residues from the cellular protein content, without disrupting cellular growth (Lyons et al., 2013). Additionally, the high stability of this compound grants labeling analysis for several months, allowing the tracking of human cellular division for eight or more generations (Luzyanina et al., 2013). These characteristics make CFSE labeling a valuable tool of cell cycle dynamics using fluorescence microscopy and cytometry (Tuominen-Gustafsson et al., 2006). In fact, other techniques commonly used to monitor cell cycle, such as bromodeoxyuridine and tritiated thymidine incorporation, present limited applications—the former technique, for instance, is only feasible in situations in which cells undergo a limited number of replication events; the latter, precludes the analysis of single cell proliferation (Lyons et al., 2013).

During each cycle of cellular duplication, CFSE fluorescence intensity decreases to the half of its initial median, allowing the detection of newly-generated cells (Lyons and Parish, 1994). In addition, our group observed that arrested-cells (obtained by the exposure to 500 Gy of gamma radiation) show a slight reduction in CFSE fluorescence intensity after 24 h of culture growth, although this intensity decrease is not related to a replicational event (data not shown). This small decay in CFSE labeling may be due to the instability of covalent bonds established between lysine residues and CFSE, as previously reported in lymphocytes (Parish et al., 2009). For this reason, we considered the time point of 24 h of culture growth as the maximum value of CFSE fluorescence intensity for all analyses. Thus, since replication is expected to promote a 0.55-fold drop in total CFSE fluorescence intensity by each replication cycle, *T. cruzi* cells which maintained CFSE fluorescence intensity above the median of CFSE fluorescence intensity observed at 24 h of cellular growth were considered as dormant cells.

Based on the aforementioned conditions, we showed that 24 h of culture growth is enough to observe cellular replication in all strains tested. Additionally, there is a percentage of epimastigotes that do not duplicate during 144 h of culture growth, indicating cell arrest, which characterizes a dormancy state in this stage of *T. cruzi'*s life cycle. Also, we observed that non-hybrid strains—i.e., Dm28c and Y—exhibit higher replication rate than naturally-occurring hybrid strains—CL Brener and Bug. In fact, Dm28c and Y reached the stationary phase of growth earlier than CL Berner and Bug. These results correlate well with the percentage of cells that did not undergo replication after 144 h of cellular culture. While naturally-occurring hybrid strains showed accumulation of arrested cells through the 144 h time interval, characterizing an asynchronous replication pattern, non-hybrid strains went opposite ways, with a higher and more synchronized duplication rate. Indeed, the decay of CFSE fluorescence intensity over time supports this conclusion as CL Brener and Bug showed a wider distribution of CFSE labeling when compared to Dm28c and Y, certainly due to their asynchronous replication cell cycle.

In order to further investigate whether arrested *T. cruzi* parasites still kept the ability to replicate, we set out an isolation protocol using FACSAria to generate a culture enriched in dormant epimastigotes. We were able to obtain a culture of Bug epimastigotes enriched in 1,550%, and observed that the majority (96%) of arrested epimastigotes can resume cellular replication once in fresh LIT medium, while 4% from total epimastigotes remained arrested. Dumoulin and Burleigh (2018), through the use of CFSE to monitor amastigote replication, showed the existence of a replicative plasticity of *T. cruzi* under nutritional depletion. It is possible that, under those conditions, *T. cruzi* arrested cells were still able to replicate, but the environmental conditions—i.e., culture reaching the stationary phase and lack of nutrients in the culture medium—may have prevented cellular replication. In fact, the addition of fresh culture medium could be a signal for the replication recovery we observed in our conditions.

As cited above, even in enriched cultures of dormant Bug epimastigotes, we still found a percentage of parasites (4%) that did not undergo replication after being resuspended in fresh LIT. Interestingly, this percentage corresponds to the percentage of dormant epimastigotes present in the original Bug epimastigote culture before sorting. These results may indicate that induction of dormancy in a defined percentage of cells may be an intended process, which acts in parallel with replication recovery promoted by fresh LIT. In this context, in which extracellular molecular signaling appears to play a role in the cellular fate of a given organism, we hypothesize that a mechanism that can resemble quorum-sensing may be determining this phenotype in *T. cruzi*. In fact, quorum-sensing has been observed in other trypanosomatids such as *Trypanosoma brucei* (Mony and Matthews, 2015). In this parasite, a molecule called stumpy induction factor (SIF) is capable of retarding parasite growth, in a density-dependent way, when it is cultured *in vitro*. A percentage from total parasite population, however, does not respond to the molecule, allowing asynchronous growth. SIF is already shown to modulate the expression of proteins involved in homologous recombination in *T. brucei* (Mony and Matthews, 2015). Besides that, arrested/dormant cells are observed for a number of other organisms: fungal dormant spores seem to avoid germination in harmful conditions through a very well-regulated way (Wyatt et al., 2013); in bacteria, the generation of dormant cells is stimulated in a way that perseveration of host colonization can be conducted in the case of a catastrophic event like antibiotic exposure (Harms et al., 2016); in protozoa, the arrested/dormant state has already been characterized as a specific form of *Plasmodium* sp. called hypnozoite—hypnozoites have been associated with the recurrence of malaria, as well as with drug resistance during treatment of this disease (Markus, 2012). In addition, cancerous cells also present a dormant state, which helps escaping chemotherapy, aimed to kill replicatively-active cells (Naumov et al., 2001).

We then went on to further investigate whether dormancy could be also observed in *T. cruzi* amastigotes. For such, we infected LLC-MK2 cells with CFSE pre-labeled trypomastigotes, and followed CFSE fluorescence intensity over time. CFSE-positive amastigotes found after 96 h of infection were considered arrested. First, it was possible to observe that hybrid strains—CL Brener and Bug—were less infective than non-hybrid strains—DM28c and Y—corroborating previous data from Zingales et al. (1997). Second, CL Brener and Bug strains exhibited the highest percentages of arrested amastigotes, showing a negative correlation between dormancy and infectivity in *T. cruzi*.

Considering the intracellular replication of *T. cruzi*, non-hybrid strains DM28c and Y presented an increased rate of replication cycle when compared to naturally-occurring hybrid CL Brener and Bug ones, which is in line with their virulence in mice (Medeiros et al., 2010) and in LLC-MK2 cells (Zingales et al., 1997). In fact, *T. cruzi*'s virulence also correlates with the percentage of arrested cells found in those strains. Dm28c and Y present high replication rates, low dormancy and increased virulence, while CL Brener and Bug exhibit low replication rates, high dormancy and decreased virulence. In addition, Dm28c and CL Brener also displayed a reduction in the average number of amastigotes present in LLC-MK2 cells from 72 to 96 h post-infection (Figure 3B). It is likely that these reduction in the average number of intracellular parasites per infected cell is due to parasite reinfection events, since for these cultures, although the proportion of cells containing a single amastigote drops 48 h post-infection it rises again at 96 h post-infection. On the other hand, the percentage of LLC-MK2 cells infected by a single *T. cruzi* amastigote from Y and Bug strains does not rise at 96 h post-infection. We further verified that the aforementioned reinfection process is performed efficiently by CFSE-positive parasites, since a significant proportion of these reinfection events occurred by CFSE positive parasites, more than 15% for CL Brener and 40% for DM28c strain. When we consider that the percentage of CFSE-positive cells 72 h post-infection was around 0.5 and 2% for Dm28c and CL Brener, respectively, we can assume that arrested amastigotes not only present the ability to differentiate into new trypomastigotes (Sánchez-Valdéz et al., 2018), but also exhibit increased infectivity in relation to non-arrested parasites. One possible reason by which they would be more infective relies on the possibility that the earlier one *T. cruzi* becomes arrested, the lesser changes would be observed in its surface proteins in relation to the parental amastigote. Therefore, *T. cruzi* cells presenting high infection ability would be selected to perpetrate further waves of reinfection in the host. This scenario could be a valuable strategy for *T. cruzi* to establish the chronic phase of Chagas disease, since these arrested parasites would partially halt host-cell colonization, reducing the number of trypomastigotes released to the extracellular milieu, thus decreasing parasite detection by the host immune system. This hypothesis would be in accordance with the clinically-observed patterns of Chagas disease progression and strain-dependent chronicity, in which naturally-hybrid strains show higher rates of chronification (Oliveira et al., 2017). On the other hand, *T. cruzi* strains that present less percentage of arrested cells would be less prone to establish a chronic infection. In fact, there is no reinfection process performed by CFSE-positive parasites when LLC-MK2 cells are infected by the Y strain (data not shown). Interestingly, data from experimental infection in mice show that animals exhibit high parasitemia within few days of infection with the Y strain, which usually leads to death, with few cases of chronicity (Zingales et al., 2012). On the contrary, infection with *T. cruzi* from CL Brener strain usually leads to chronification during experimental infection in mice (Medeiros et al., 2010).

Sánchez-Valdéz et al. (2018) hypothesized that homologous recombination is the cause for dormancy in *T. cruzi*. It is well-known that the recombination pivot is TcRad51, a protein responsible for the repair of DSBs—either induced by high levels of gamma radiation, or caused by other genotoxic agents that lead to transcriptional and replicational fork arrest (Regis-da-Silva et al., 2006; Gomes Passos Silva et al., 2018). We then observed the effect of gamma radiation in inducing arrest in *T. cruzi* epimastigotes. Gamma rays, as previously described (Regis-da-Silva et al., 2006; Alves et al., 2018; Gomes Passos Silva et al., 2018), prevented parasite replication for 72 h, with growth recovery being observed after 96 h (Figure 5A). Despite replication recovery, at 144 h of culture, the percentage of dormant cells was still higher in irradiated cultures when compared to non-irradiated ones, corroborating the role of homologous recombination in inducing arrested cells. In fact, transcriptional levels of TcRAD51 from the strains used in this study led us to establish a correlation between the rate of arrested parasites and the relative level of TcRAD51 mRNA. As previously shown by Alves et al. (2018), naturally-occurring hybrid strains displayed increased transcription rates of TcRAD51 when compared to non-hybrid ones, showing that the higher the transcription of TcRAD51, the higher the number of arrested cells. Altogether, these observations reinforce the role of homologous recombination process in the induction dormancy in *T. cruzi*.

In order to check, the importance of homologous recombination in *T. cruzi* dormancy, we next investigated the percentage of dormant amastigotes and infectivity of a *T. cruzi* mutant from strain CL Brener which lacks one of the two copies of TcRAD51 (CL Brener^RAD51+/−^), which was previously characterized by our group (Gomes Passos Silva et al., 2018). CL Brener^RAD51+/−^ epimastigotes showed a reduction in the number of arrested cells without growth impair (Figures 7A,B). These observations corroborated the direct relationship between homologous recombination and dormancy in *T. cruzi*.

Interestingly, CL Brener^RAD51+/−^ trypomastigotes also exhibited reduced infectivity. Previous data from our group showed that, in Dm28c strain, the complete depletion of TOPO3α gene—which encodes Topo3α, an enzyme responsible for the resolution of recombination products (Capranico et al., 2017)—was similarly able to reduce parasite infection rate (unpublished data).

Altogether, our data show that the ability to enter in an arrested state is a strain-dependent phenomenon in *T. cruzi*, and that homologous recombination may play a role in this process. Additional studies are needed to better characterize the biology of the arrested cells—both epimastigotes and amastigotes—in this organism and the function of recombination in the induction of dormancy.

## Data Availability Statement

The raw data supporting the conclusions of this article will be made available by the authors, without undue reservation, to any qualified researcher.

## Author Contributions

BCR conceptualized the study, carried out the formal analysis and investigation, and wrote the original draft of the article. CM conceptualized the study, carried out the formal analysis, and wrote the article. AO, AG, VS, and PH carried out the investigation and formal analysis. BMR carried out the investigation and formal analysis, and wrote the article. SP acquired funding and resources. GF and AM carried out the formal analysis, and acquired funding and resources. ET carried out the formal analysis, acquired funding and resources, and wrote the article. SF carried out the formal analysis, wrote the methodology, and conceptualized the study. LA and CM conceptualized the study, carried out the formal analysis and investigation, wrote the article, acquired funding and resources, and wrote the methodology.

### Conflict of Interest

The authors declare that the research was conducted in the absence of any commercial or financial relationships that could be construed as a potential conflict of interest.

## References

[B1] Alves C. L. Repolês B. M. da Silva M. S. Mendes I. C. Marin P. A. Aguiar P. H. N.. (2018). The recombinase Rad51 plays a key role in events of genetic exchange in *Trypanosoma cruzi*. Sci. Rep. 8, 1–12. 10.1038/s41598-018-31541-z 30190603 PMC6127316

[B2] Andrade L. O. Andrews N. W. (2004). Lysosomal fusion is essential for the retention of *Trypanosoma cruzi* inside host cells. J. Exp. Med. 200, 1135–1143. 10.1084/jem.20041408 15520245 PMC2211867

[B3] Andrade L. O. Andrews N. W. (2005). Opinion: the *Trypanosoma cruzi*- host-cell interplay: location, invasion, retention. Nat. Rev. Microbiol. 3, 819–823. 10.1038/nrmicro1249 16175174

[B4] Andrews N. W. Hong K. S. Robbins E. S. Nussenzweig V. (1987). Stage-specific surface antigens expressed during the morphogenesis of vertebrate forms of *Trypanosoma cruzi*. Exp. Parasitol. 64, 474–484. 10.1016/0014-4894(87)90062-2 3315736

[B5] Brenière S. F. Waleckx E. Barnabé C. (2016). Over six thousand *Trypanosoma cruzi* strains classified into discrete typing units (DTUs): attempt at an inventory. PLoS Negl. Trop. Dis. 10, 1–19. 10.1371/journal.pntd.0004792 27571035 PMC5003387

[B6] Capranico G. Marinello J. Chillemi G. (2017). Type I DNA topoisomerases. J. Med. Chem. 60, 2169–2192. 10.1021/acs.jmedchem.6b00966 28072526

[B7] De Souza W. (1999). A short review on the morphology of *Trypanosoma cruzi*: from 1909 to 1999. Mem. Inst. Oswaldo Cruz 94, 17–36. 10.1590/S0074-02761999000700003 10677689

[B8] Dumoulin P. C. Burleigh B. A. (2018). Stress-induced proliferation and cell cycle plasticity of intracellular *Trypanosoma cruzi* amastigotes. Mbio 9, e00673–e00618. 10.1128/mBio.00673-18 29991586 PMC6050952

[B9] Fernandes M. C. Andrews N. W. (2012). Host cell invasion by *Trypanosoma cruzi:* a unique strategy that promotes persistence. FEMS Microbiol. Rev. 36, 734–747. 10.1111/j.1574-6976.2012.00333.x 22339763 PMC3319478

[B10] Gomes Passos Silva D. da Silva Santos S. Nardelli S. C. Mendes I. C. Freire A. C. G. Repolês B. M.. (2018). The *in vivo* and *in vitro* roles of *Trypanosoma cruzi* Rad51 in the repair of DNA double strand breaks and oxidative lesions. PLoS Negl. Trop. Dis. 12:e0006875. 10.1371/journal.pntd.0006875 30422982 PMC6258567

[B11] Harms A. Maisonneuve E. Gerdes K. (2016). Mechanisms of bacterial persistence during stress and antibiotic exposure. Science 354:aaf4268. 10.1126/science.aaf4268 27980159

[B12] Holloman W. K. (2011). Unraveling the mechanism of BRCA2 in homologous recombination. Nat. Struct. Mol. Biol. 18, 748–754. 10.1038/nsmb.2096 21731065 PMC3647347

[B13] Hull R. N. Cherry W. R. Tritch O. J. (1962). Growth characteristics of monkey kidney cell strains LLC-Mk1, LLC-Mk2, and LLC-MK2(NCTC-3196) and their utility in virus research. J. Exp. Med. 115, 903–918. 10.1084/jem.115.5.903 14449901 PMC2137539

[B14] Luzyanina T. Cupovic J. Ludewig B. Bocharov G. (2013). Mathematical models for CFSE labelled lymphocyte dynamics: asymmetry and time-lag in division. J. Math. Biol. 69, 1547–1583. 10.1007/s00285-013-0741-z 24337680

[B15] Lyons A. B. Blake S. J. Doherty K. V. (2013). Flow cytometric analysis of cell division by dilution of CFSE related dyes. Curr. Protoc. Cytom. Chapter 9:Unit 9.11. 10.1002/0471142956.cy0911s64 23546777

[B16] Lyons A. B. Parish C. R. (1994). Determination of lymphocyte division by flow cytometry. J. Immunol. Methods 171, 131–137. 10.1016/0022-1759(94)90236-4 8176234

[B17] Markus M. B. (2012). Dormancy in mammalian malaria. Trends Parasitol. 28, 39–45. 10.1016/j.pt.2011.10.005 22118814

[B18] Medeiros M. Araújo-Jorge T. C. Batista W. S. Da Silva T. M. O. A. De Souza A. P. (2010). *Trypanosoma cruzi* infection: do distinct populations cause intestinal motility alteration? Parasitol. Res. 107, 239–242. 10.1007/s00436-010-1871-5 20454805

[B19] Mony B. M. Matthews K. R. (2015). Assembling the components of the quorum sensing pathway in African trypanosomes. Mol. Microbiol. 96, 220–232. 10.1111/mmi.12949 25630552 PMC4403954

[B20] Naumov G. N. MacDonald I. C. Chambers A. F. Groom A. C. (2001). Solitary cancer cells as a possible source of tumour dormancy? Semin. Cancer Biol. 11, 271–276. 10.1006/scbi.2001.0382 11513562

[B21] Oliveira T. D. dos Santos B. N. Galdino T. S. Hasslocher-Moreno A. M. Bastos O. M. P. Sousa M. A. (2017). *Trypanosoma cruzi* i genotype among isolates from patients with chronic chagas disease followed at the evandro chagas national institute of infectious diseases (FIOCRUZ, Brazil). Rev. Soc. Bras. Med. Trop. 50, 35–43. 10.1590/0037-8682-0406-2016 28327800

[B22] Parish C. R. Glidden M. H. Quah B. J. C. Warren H. S. (2009). Use of the intracellular fluorescent dye CFSE to monitor lymphocyte migration and proliferation. Curr. Protoc. Immunol. Chapter 4: Unit 4.9. 10.1002/0471142735.im0409s84 19235770

[B23] Ragone P. G. Brandán C. P. Rumi M. M. Tomasini N. Lauthier J. J. Cimino R. O.. (2015). Experimental evidence of biological interactions among different isolates of *Trypanosoma cruzi* from the Chaco Region. PLoS ONE 10:e0119866. 10.1371/journal.pone.0119866 25789617 PMC4366099

[B24] Rassi A. Jr. Marin-Neto A. (2010). Seminar Chagas disease. Lancet 375, 1388–1402. 10.1016/S0140-6736(10)60061-X 20399979

[B25] Regis-da-Silva C. G. Freitas J. M. Passos-Silva D. G. Furtado C. Augusto-Pinto L. Pereira M. T.. (2006). Characterization of the *Trypanosoma cruzi* Rad51 gene and its role in recombination events associated with the parasite resistance to ionizing radiation. Mol. Biochem. Parasitol. 149, 191–200. 10.1016/j.molbiopara.2006.05.012 16828179

[B26] Sánchez-Valdéz F. J. Padilla A. Wang W. Orr D. Tarleton R. L. (2018). Spontaneous dormancy protects *Trypanosoma cruzi* during extended drug exposure. Elife 7, 1–20. 10.7554/eLife.34039 29578409 PMC5906098

[B27] Tomasini N. Diosque P. (2015). Evolution of *Trypanosoma cruzi*: clarifying hybridisations, mitochondrial introgressions and phylogenetic relationships between major lineages. Mem. Inst. Oswaldo Cruz 110, 403–413. 10.1590/0074-02760140401 25807469 PMC4489478

[B28] Tuominen-Gustafsson H. Penttinen M. Hytönen J. Viljanen M. K. (2006). Use of CFSE staining of borreliae in studies on the interaction between borreliae and human neutrophils. BMC Microbiol. 6:92. 10.1186/1471-2180-6-92 17049082 PMC1621068

[B29] Vago A. R. Andrade L. O. Leite A. A. d'Ávila Reis D. Macedo A. M. Adad S. J.. (2000). Genetic characterization of *Trypanosoma cruzi* directly from tissues of patients with chronic chagas disease: differential distribution of genetic types into diverse organs. Am. J. Pathol. 156, 1805–1809. 10.1016/S0002-9440(10)65052-3 10793092 PMC1876933

[B30] WHO (2019). Chagas Disease (American Trypanosomiasis). WHO. Available online at: https://www.who.int/chagas/en/ (accessed September 25, 2019).

[B31] Wyatt T. T. Wösten H. A. B. Dijksterhuis J. (2013). Fungal Spores for Dispersion in Space and Time, 1st Edn. Utrecht: Elsevier Inc 10.1016/B978-0-12-407672-3.00002-2 23942148

[B32] Zingales B. (2018). *Trypanosoma cruzi* genetic diversity: something new for something known about Chagas disease manifestations, serodiagnosis and drug sensitivity. Acta Trop. 184, 38–52. 10.1016/j.actatropica.2017.09.017 28941731

[B33] Zingales B. Andrade S. G. Briones M. R. S. Campbell D. A. Chiari E. Fernandes O.. (2009). A new consensus for *Trypanosoma cruzi* intraspecific nomenclature: second revision meeting recommends TcI to TcVI. Mem. Inst. Oswaldo Cruz 104, 1051–1054. 10.1590/S0074-02762009000700021 20027478

[B34] Zingales B. Miles M. A. Campbell D. A. Tibayrenc M. Macedo A. M. Teixeira M. M. G.. (2012). The revised *Trypanosoma cruzi* subspecific nomenclature: rationale, epidemiological relevance and research applications. Infect. Genet. Evol. 12, 240–253. 10.1016/j.meegid.2011.12.009 22226704

[B35] Zingales B. Pereira M. E. S. Almeida K. A. Umezawa E. S. Nehme N. S. Oliveira R. P.. (1997). Biological parameters and molecular markers of clone CL brener - the reference organism of the *Trypanosoma cruzi* genome project. Mem. Inst. Oswaldo Cruz 92, 811–814. 10.1590/S0074-02761997000600016 9566213

